# Evaluating the estimation of genetic correlation and heritability using summary statistics

**DOI:** 10.1007/s00438-021-01817-7

**Published:** 2021-09-29

**Authors:** Ju Zhang, Fredrick R. Schumacher

**Affiliations:** Department of Population and Quantitative Health Sciences, Case Western Reserve University, Cleveland, OH 44106 USA

**Keywords:** Genetic correlation, Heritability, Summary statistics, Evaluation

## Abstract

While novel statistical methods quantifying the shared heritability of traits and diseases between ancestral distinct populations have been recently proposed, a thorough evaluation of these approaches under differing circumstances remain elusive. Brown et al.^2016^ proposed the method *Popcorn* to estimate the shared heritability, *i.e.* genetic correlation, using only summary statistics. Here, we evaluate *Popcorn* under several parameters and circumstances: sample size, number of SNPs, sample size of external reference panel, various population pairs, inappropriate external reference panel, and admixed population involved. Our results determined the minimum sample size of the external reference panel, summary statistics, and number of SNPs required to accurately estimate both the genetic correlation and heritability. Moreover, the number of individuals and SNPs required to produce accurate and stable estimates was directly proportional with heritability in *Popcorn.* Misrepresentation of the reference panel overestimated the genetic correlation by 20% and heritability by 60%. Lastly, applying *Popcorn* to homogeneous (EUR) and admixed (ASW) populations underestimated the genetic correlation by 15%. Although statistical approaches estimating the shared heritability between ancestral populations will provide novel etiologic insight, caution is required ensuring results are based on the appropriate sample size, number of SNPs, and the generalizability of the reference panel to the discovery populations.

**Supplementary Information:**

The online version contains supplementary material available at 10.1007/s00438-021-01817-7.

## Introduction

As the number of genome-wide association studies conducted in both single- and multi-ancestral populations increase, the significance of estimating genetic correlation for complex traits has become more enormous. Brown et al. 2016 expanded the definition of genetic correlation to the trans-ancestral context and developed the method *Popcorn* to estimate genetic correlation using summary statistics. Although *Popcorn* produces unbiased estimators of genetic correlation and heritability, it requires large sample sizes, large number of SNPs and matched external reference panels, and is limited to the utility of homogenous populations. We evaluated *Popcorn* under several parameters and circumstances: sample size, number of SNPs, sample size of external reference panel, various population pairs, inappropriate external reference panel and admixed population involved. It provides plenty of valuable reference information for researchers using the method to obtain more accurate and stable estimates for both genetic correlation and heritability.

Although the causes of many complex diseases are not yet fully understood, genetic variation clearly impacts the susceptibility of diseases such as prostate (Schaid 2004), breast (Black 1994) and type 2 diabetes (Yukio Horikawa 2000). A large twin study reported that the heritability of cancer overall is 33% (95% CI 30–37%) and significant heritability is observed for several cancer types such as melanoma (58%; 95% CI 43–73%), prostate (57%; 95% CI 51–63%), nonmelanoma skin (43%; 95% CI 26–59%), ovary (39%; 95% CI 23–55%), kidney (38%; 95% CI 21–55%), breast (31%; 95% CI 11–51%), and corpus uteri (27%; 95% CI 11–43%) in Nordic countries (Mucci et al. 2016). In recent years, numerous genome-wide association studies (GWAS) have been conducted focusing on identifying the genetic architecture of these diseases. However, the vast majority of GWAS have been conducted in individuals of European ancestry with much less focus on non-European and multi-ancestral populations. This has led to a gap elucidating the genetic contribution in non-European populations, including the overall shared heritability between ancestral populations.

Therefore, estimating *shared heritability*, the measure of the common genetic contribution for two traits or a common trait between two ancestral populations, can provide important information for the transferability of epidemiological results. For example, the known genetic architecture of a trait in population A will transfer to population B if the shared heritability of the trait is substantial between populations A and B. On the contrary, we will not expect the same results on B if the shared heritability is close to 0. Estimating shared heritability could facilitate mapping the causative loci for diseases in multiple ethnic population with information from GWAS studies (Coram et al. 2015; Morris 2011).

Genetic correlation is a component of shared heritability and indicates the extent we can treat ancestral populations as one regarding the underlying disease genetic architecture. Furthermore, shared heritability has profound implications in the biomedical and clinical practice of diverse populations, assessing the contribution of non-additive and rare-variant effects, and modeling the genetic architecture of complex traits (Brown et al. 2016). Quantifying the genetic correlation of a complex trait between ancestral distinct populations may be instrumental identifying underlying determinants of health disparities.

Genetic correlation can be estimated from genotype- and mixed-model-based methods available in several software packages. For example, GCTA is able to perform a bivariate restricted maximum likelihood analysis to estimate the genetic correlation between two quantitative traits, two binary disease traits, or a quantitative and a binary disease trait (Lee et al. 2012; Yang et al. 2011). However, these approaches require individual-level genetic data where access may raise privacy concerns or additional logistical considerations thus restricting access to individual-level data (refers to genome-wide single nucleotide polymorphisms (SNP) and trait values for each individual included in a GWAS. However, summary associations are not scaled by the number of individuals in a study and often are readily accessible Benner C et al. 2017. The recent public availability of summary statistics from GWAS meta-analyses has generated exciting new opportunities to develop statistical methods without access to individual-level genotype–phenotype data (Benner et al. 2017). For example, linkage disequilibrium (LD) score regression has been developed to estimate heritability and genetic correlation from GWAS summary statistics between two traits through regressing *χ*^2^ statistics against LD scores generated in a reference population (Bulik-Sullivan et al. 2015a, b). However, this method is limited to a single homogenous population (Bulik-Sullivan et al. 2015b).

Brown et al. (2016) expanded the definition of genetic correlation to better account for a trans-ancestral context and developed the novel approach *Popcorn,* where estimating genetic correlation between differing ancestral populations using only summary association statistics is feasible. GWAS summary statistics refer to estimated effect sizes and their standard errors (SE) for each SNP analyzed and sometimes include the allele frequency. *Popcorn* considers both allele effect sizes and allelic impact to define trans-ancestral genetic-effect correlation as correlation coefficient of the per-allele SNP effect sizes (Lee et al. 2012) and trans-ancestral genetic-impact correlation as the correlation coefficient of the population-specific allele-variance-normalized SNP effect sizes (Brown et al. 2016). The method combines the information of LD from external reference panels with summary association data to avoid privacy concerns while using the entire spectrum of GWAS associations accounting for LD to avoid filtering correlated SNPs (Brown et al. 2016) employed a Bayesian approach wherein they assume genotypes are drawn separately from within each population and effect sizes have a normal prior. The infinitesimal assumption yields a multivariate normal distribution on the observed test statistics, where the covariance matrix is a function of the heritability and genetic correlation. Then they maximized an approximate weighted likelihood function to find the heritability and genetic correlation (Brown et al. 2016).

*Popcorn* has been utilized on several occasions, including European and Yoruban gene-expression data (Hoen et al. 2013), GWAS summary statistics from European and East Asian cohorts with rheumatoid arthritis (RA) and type 2 diabetes (T2D), and Genetic European Variation in Health and Disease (GEUVADIS) data (Chang et al. 2015; Su et al. 2011). As demonstrated in Brown et al. 2016, the method produced unbiased estimates of the genetic correlation and the population-specific heritability with a standard error (SE) inversely correlated with the number of SNPs and individuals in the studies (Brown et al. 2016). The summary statistic-based estimator was shown to be concordant with the mixed-model-based estimator (Brown et al. 2016). Although *Popcorn* is powerful, it still has several limitations: (1) a large sample size and number of SNPs is required to achieve a sufficient SE for accurate estimation, (2) limited to relatively common SNPs with allele frequency greater than 0.05, and 3) relies on LD similarity between the external reference panels and the target populations.

Although the application of *Popcorn* to estimate genetic correlation across differing ancestral populations is powerful, potential limitations include a thorough evaluation of *Popcorn*. First, it is important to define a ‘large’ sample size and the number of SNPs to better interpret results when applied to summary statistics. Different sample sizes are available for different traits and include a wide range. For example, summary statistics of 184,035 individuals are available for coronary artery disease (Nikpay et al. 2015) while only 5422 for attention deficit hyperactivity disorder Neale et al. (2010) among individuals of European ancestry. A greater sample size discrepancy will exist when evaluating non-European ancestral populations. The paper fails to provide a specific minimum number large enough for accuracy though it tests 5,000, 10,000, and 15,000 when using simulated data and 20,000–60,000 individuals for an applied example between European and East Asian populations. Second, it is necessary to find the smallest sample size of the external reference panel where the minimum accuracy of the estimates is lost from the analysis given different sample sizes of summary statistics. Brown et al. 2016 uses about 500 individuals from each ancestrally distinct population to serve as the external reference panel for both simulation and real data tests. Obviously, a larger number of individuals in the reference panel can better capture the LD structure of the target population, particularly for lower allele frequencies (MAF < 0.05). However, the number of individuals is directly correlated with the required computational time since LD score is calculated from individual-level data. Furthermore, for some population pairs, the sample sizes of the reference panel available might be very different while the complexity of the LD structure for different populations may be inconsistent. If we want to use data from the 1000 Genome project, for example, as the reference panel, there are 504 and 503 individuals in the EUR and EAS populations while only 62 and 120 in ASW and YRI, respectively (Genomes Project et al. 2015). The accuracy of the estimation from *Popcorn* under such circumstance is also expected to be tested. Third, the frequency threshold for the minor allele will compromise the accuracy of the estimation. The allele frequency range should be determined for application of *Popcorn*. Fourth, the performance of *Popcorn* on other ancestral population (such as African) other than European and East Asian should also be evaluated. All of the previous simulations were performed on Europeans and East Asians and the only two real data applications focused on RA and T2D are also on these two populations and obtained relatively underestimated results due to, as explained in the paper, the correction of genomic control in summary association data (Brown et al. 2016). It is still not clear how *Popcorn* performs if other ancestral population (such as African) involved when the test parameters (sample size, number of SNPs, minor allele frequency, etc.) change. Lastly, it is instructive to check how *Popcorn* will perform if we use an inappropriate reference panel. External reference panels are the only resource to provide the information of LD matrix while we do not have individual-level data of target populations. The similarity of LD structure between reference panel and target population determines the accuracy of following estimation. It is very important to choose an appropriate external reference panel during the analysis process in *Popcorn* while sometimes the structure of the target population is unclear.

## Materials and methods

*Popcorn* utilizes summary association statistics from differing ancestral populations as input and LD based on external reference panels similar to the populations. It first estimates the LD matrix products then maximizes an approximate weighted likelihood function to find heritability and genetic correlations, respectively (Brown et al. 2016). Here to obtain simulated data to perform the evaluation, we simulated European (EUR), East Asian (EAS), and Yoruban (YRI) ancestral populations based on HapMap3 data. Furthermore, we simulated an admixed African ancestry based on southwest USA (ASW) populations from HapMap 3. We generated ${N}_{1}$ and ${N}_{2}$ individuals for population 1 (pop1) and 2 (pop2), respectively, in each population pair; and $M$ SNPs after filtering rare alleles with minor allele frequency below ${f}_{0}$ and relatedness of individuals above ${r}_{0}$. We randomly selected $L$ individuals from each population as an external reference panel. Effect sizes for SNP$i$, ${\beta }_{i}=({\beta }_{1i},{\beta }_{2i})$ ^T^, are drawn from a ‘spike and slab’ model,

$$\beta _{{1i}} ,\beta _{{2i}} \left\{ {\begin{array}{*{20}c} {\sim N\left( {\left[ {\begin{array}{*{20}c} 0 \\ 0 \\ \end{array} } \right],\left[ {\begin{array}{*{20}c} {\frac{{h_{1}^{2} }}{M}} & {{\text{amp}};\frac{{\rho _{{{\text{ge}}}} \sqrt {h_{1}^{2} h_{2}^{2} } }}{M}} \\ {\frac{{\rho _{{{\text{ge}}}} \sqrt {h_{1}^{2} h_{2}^{2} } }}{M}} & {{\text{amp}};\frac{{h_{2}^{2} }}{M}} \\ \end{array} } \right]} \right),{\text{with probability }}p} \\ { = \left( {0,0} \right),{\text{with probability }}1 - p,} \\ \end{array} } \right.,$$

where ${h}_{1}^{2}$ and ${h}_{2}^{2}$ is heritability of pop1 and pop2, respectively. Let ${f}_{1}$ and ${f}_{2}$ be the allele frequency in pop1 and pop2 and ${\rho }_{\mathrm{gi}}$ is calculated from $\beta$ as

$${\rho }_{\mathrm{gi}}=\mathrm{Cor}\left({\sigma }_{1}{\beta }_{1}, {\sigma }_{2}{\beta }_{2}\right),$$

where ${\sigma }_{1}=\sqrt{2{f}_{1}(1-{f}_{1})}$, ${\sigma }_{2}=\sqrt{2{f}_{2}(1-{f}_{2})}$, and $Cor$ refers to correlation. The phenotypes of pop1 ${Y}_{1}$ are simulated from an additive genetic model

$${Y}_{1}=\sum_{i}{\upbeta }_{1i}{X}_{1i}+ {\upvarepsilon }_{1,}$$

where ${X}_{1}$ is normalized genotypes and ${\varepsilon }_{1}$ is the residual effect generated from a normal distribution with mean of 0 and variance of $1-{h}_{1}^{2}$. We vary population pairs, ${N}_{1}$, ${N}_{2}$, $\mathrm{M}$, ${\mathrm{f}}_{0}$, $\mathrm{L}$, ${h}_{1}^{2}$, ${h}_{2}^{2},$ and ${\rho }_{\mathrm{ge}}$ to obtain different external reference panels and summary statistics so that we could test the performance of *Popcorn*. We define ‘ideal estimates’ as the results with ‘accurate’ (bias is less than 0.05) and ‘stable’ (SE is less than 0.1) estimates.

## Results

### Reproducibility of simulation in Popcorn paper

**Fig. 1:**
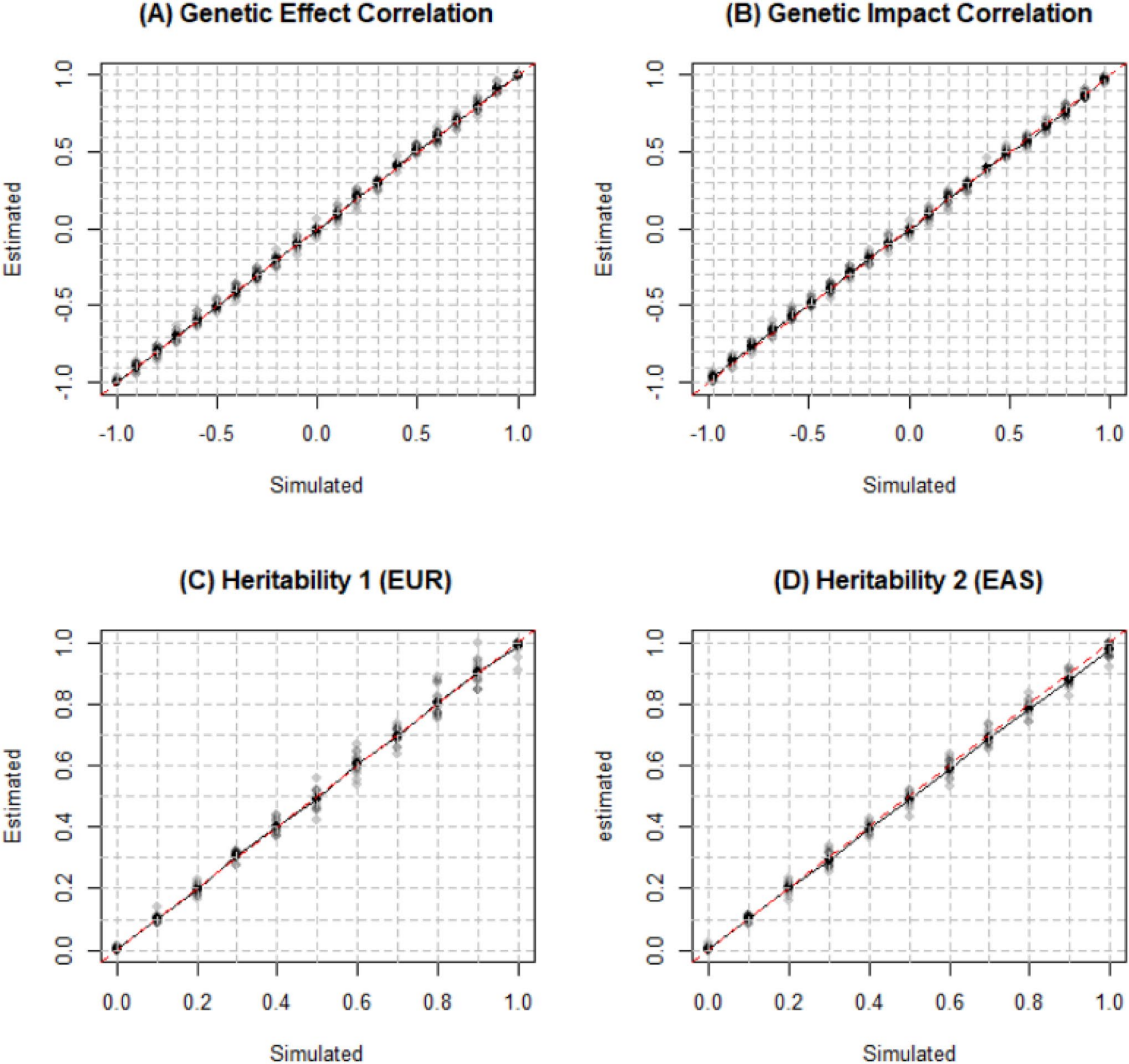
True and estimated genetic correlation and heritability for EUR and EAS. Simulations were conducted on 26,119 simulated EUR and 20,147 simulated EAS individuals with 329,382 SNPs. The default genetic correlation and heritability was 0.5

We first tried to replicate the results of simulation tests for estimation of genetic correlation and heritability in original Popcorn paper. We simulated 100,000 individuals and 329,382 SNPs from each population of EUR and EAS (Pair 1) using hapgen2 (Su et al. 2011) with an allele frequency above 1% on chromosome 1 to 3. After filtering individuals with relatedness (kinship coefficient) above 0.05, we ended with 26,119 and 20,147 individuals, respectively. Then we randomly selected 500 individuals from each population to serve as external reference panel. At last, 205,452 SNPs common in both populations after minor allele frequency and indel filter are included in the LD scores calculation. Since it has been proved that the estimators from *Popcorn* are robust to violation of infinitesimal assumption (Brown et al. 2016), we simulated effect sizes using $p=0.99$. We varied ${\rho }_{\mathrm{ge}}$ from − 1 to 1 and ${h}_{1}^{2}$ and ${h}_{2}^{2}$ from 0 to 1 at increments of 0.1 (other two parameters are 0.5 when one of them changed). Figure 1 shows observed values were very close to the expected values for genetic correlation and heritability estimated from *Popcorn*. It verified that *Popcorn* provides nearly unbiased estimation for both the genetic correlation and heritability with a slight underestimation when they are close to the boundary (Brown et al. 2016).

### Evaluation tests

#### External reference panel sample size

**Fig. 2:**
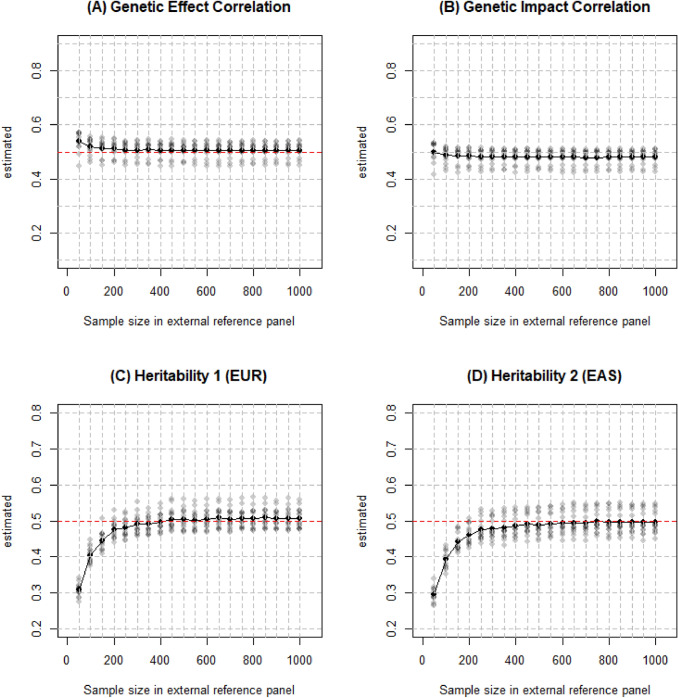
Estimated genetic correlation and heritability for EUR and EAS from different sample sizes of the external reference panel. Simulations were conducted on 26,119 simulated EUR and 20,147 simulated EAS individuals with 329,382 SNPs. The default genetic correlation and heritability was 0.5

We applied the same settings from the reproduction part but changed $L$ from 50 to 1,000 at increments of 50. Figure 2 shows that the accuracy of the estimation is increased as $L$ goes up but $L$ has little impact on the SE. As observed in the figure, a minimum of 200 people in the external reference panel are sufficient to accurately estimate the genetic correlation while a minimum of 500 are required for heritability. Furthermore, heritability of EAS requires even more people, approximately 750 in the reference panel.

#### Sample size

We varied the number of individuals though random sampling in each simulation. Without loss of generality, we made ${N}_{1}={N}_{2}=N$ in this section of simulation. We changed *N* from 1000 to 20,000 with increments of 1000. Due to the different performance *Popcorn* had for the boundary estimation, we assessed performance after setting genetic correlation and heritability both close to the boundaries of 0.1 and 0.9, and the middle of 0.5.

**Fig. 3:**
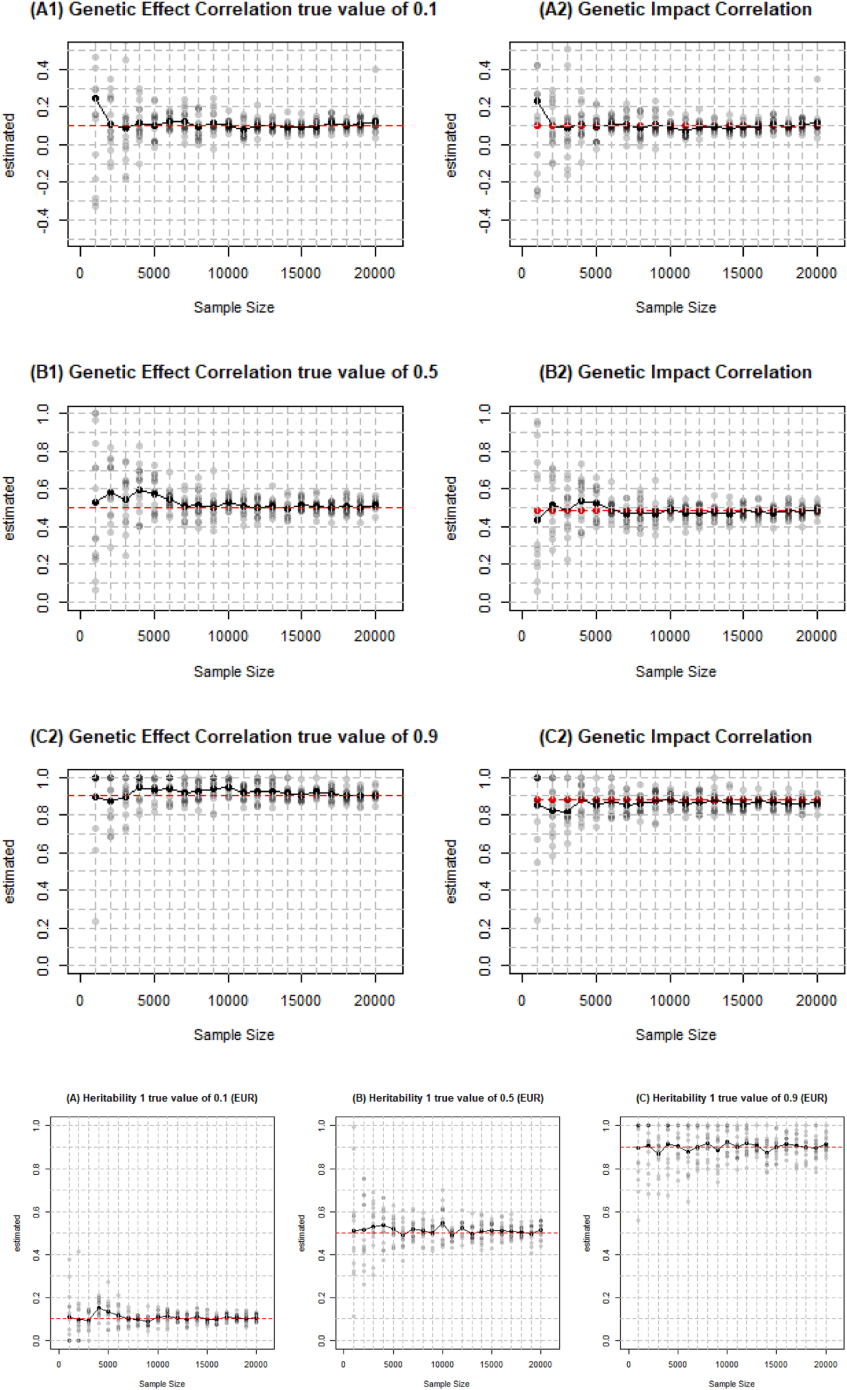
Estimated genetic correlation heritability on EUR and EAS from different sampe size. Simulations were conducted on different number of individuals with 329,382 SNPs and 500 individuals in external reference panel. The default heritability in A1 to C2 was 0.5 and genetic correlation was 0.1, 0.5, and 0.9, respectively in each row. The default genetic correlation in A, B and C was 0.5 and heritability was 0.1, 0.5, and 0.9, respectively

Figure 3A1–C2 indicates that both accuracy and stability of the estimation of the genetic correlation increased as we enlarged the sample size. The estimation of genetic correlation which is in the middle (0.5) is accurate and has small enough SE (within 0.1 up and down) when the sample size reached 10,000 and same amount of people are required for genetic correlation near the boundary (0.1 and 0.9)

Enlarging sample size lead to more accurate estimation of heritability with less SE as well (shown in Fig. 3A–C) though higher heritability generally has larger SE. Therefore, the higher the heritability is, the larger the required sample size is. We could obtain ideal estimate with 7000 individuals for heritability of 0.1, around 11,000 for 0.5 and 15,000 for 0.9.

#### Number of SNPs

We started with 205,452 SNPs on their respective chromosome and then evaluated a range of 10–100% in 10% increments on each chromosome to test different SNP densities, defined as the number of SNPs we included in the analysis over the total number of SNPs. The specific number of SNPs in each test are shown in Table 1. We show in Fig. 5 that $M$ does not have a significant impact on SE and the SE are consistently low since we have more than 20,000 individuals in the analysis. The estimation accuracy of the genetic correlation stabilizes when $M$ reaches 123,270.

**Table 1:** The density and number of SNPs in each test to evaluate Popcorn

Test #	1	2	3	4	5	6	7	8	9	10
SNP density (%)	10	20	30	40	50	60	70	80	90	100
Number of SNPs	20,543	41,089	61,634	82,180	102,725	123,270	143,815	164,361	184,906	205,452

**Fig. 4:**
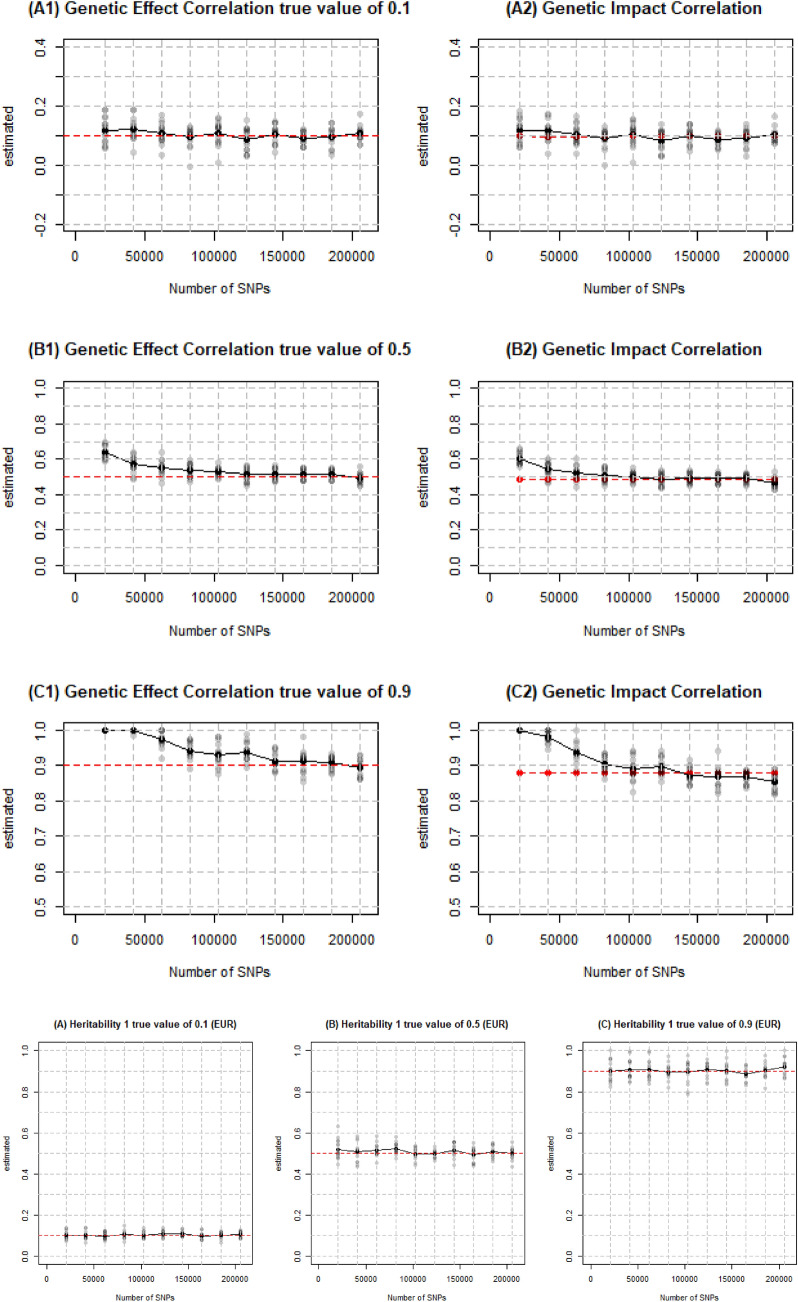
Estimated genetic correlation and heritability on EUR and EAS from different number of SNPs. Simulations were conducted on 26,119 simulated EUR and 20,147 simulated EAS individuals with different number of SNPs and 500 individuals in the external reference panel. The default heritability in A1 to C2 was 0.5 and genetic correlation was 0.1, 0.5, and 0.9, respectively in each row. The default genetic correlation in A,B and C was 0.5 and heritability was 0.1, 0.5, and 0.9, respectively

We are basically able to accurately estimate low genetic correlation when we have around 80,000 SNPs in the analysis while more than 180,000 SNPs are required to estimate high genetic correlation with an acceptable SE as shown in Fig. 4A1–C2. Overall, the higher the genetic correlation to estimate, the larger the number of SNPs required.

Figure 4A–C indicates that $\mathrm{M}$ has little effect on accuracy and stability of the estimation of heritability regardless if the true heritability lies in the middle or close to the boundary. We set $p$ as 0.99 in each test which means we set 99% of the SNPs as causal ones in the analysis no matter how many SNPs are in the analysis. Therefore, we captured all of the heritability when we adjusted $M$. In real life, however, it is usually hard to find all of causal variants and that’s also where the bias estimate of heritability usually comes from.

### Other population pairs

We did similar tests across differing ancestral population pairs, such as EUR vs. YRI (Pair 2) and EAS vs. YRI (Pair 3). The simulation steps are as same as those outlined above in reproduction part, and the detailed numbers of individuals and SNPs finally left in the two tests are shown in Table 2. We proved *Popcorn* provides unbiased estimation for genetic correlation and heritability as well (shown in Figures S1 and S7). Results from other tests which are about number of individuals in reference panel, sample size and number of SNPs are shown in Figures S2–S6 and S8–S12 and listed in Table 3 as well. First, 200–300 individuals in the external reference panel are able to provide enough information for estimates of genetic correlation while around 500–700 are needed for heritability. More specifically, *Popcorn* requires 400–500 for EUR and 700 individuals for EAS and YRI. Second, accurate genetic correlation estimates with low SE requires sample size of 10,000 for Pair 2 and 15,000 for Pair 3 and the number doesn’t change for different true genetic correlation values while at least 15,000 individuals are needed for accurate estimation of heritability with low SE and higher true heritability requires larger sample size to be accurately estimated. As for number of SNPs, more SNPs are needed for estimates of larger true genetic correlation and heritability. The required minimum number of SNPs does not have large discrepancy in either of the two population pairs. Only if we have large enough sample size, around 20,000 SNPs could be able to provide accurate estimates for heritability of 0.1 and only 90,000 are needed even for heritability of 0.9. However, genetic correlation estimates require more SNPs, ranging from 110,000 to 168,000, according to different true genetic correlation.

**Table 2:** Number of individuals and SNPs in test of EUR vs YRI and EAS vs. YRI

Populations		Individual	SNP
EUR and YRI	EUR	26,119	220,687
	YRI	35,267	
EAS and YRI	EAS	20,147	35,267
	YRI	35,267

**Table 3:** Results of minimum number of individuals in reference panel, sample size and number of SNPs for accurate and stable estimates of genetic correlation and heritability in the test of various population pairs

Population Pairs	Estimates	Values	*L*	*N*	*M*
EUR VS EAS	Genetic correlation	0.1	–	10,000	102,725
		0.5	200	10,000	123,270
		0.9	–	10,000	143,815
	Heritability	0.1	–	7,000	20,543
		0.5	500/750	11,000	41,089
		0.9	–	15,000	123,270
EUR VS YRI	Genetic correlation	0.1	–	16,000	132,411
		0.5	200	14,000	154,479
		0.9	–	16,000	154,479
	Heritability	0.1	–	7,000	22,067
		0.5	400/700	10,000	66,205
		0.9	–	15,000	88,273
EAS VS YRI	Genetic correlation	0.1	–	10,000	112,034
		0.5	300	10,000	149,379
		0.9	–	9,000	168,052
	Heritability	0.1	–	5,000	18,671
		0.5	700/700	9,000	74,689
		0.9	–	13,000	93,362

### Using inappropriate reference panel

**Fig. 5:**
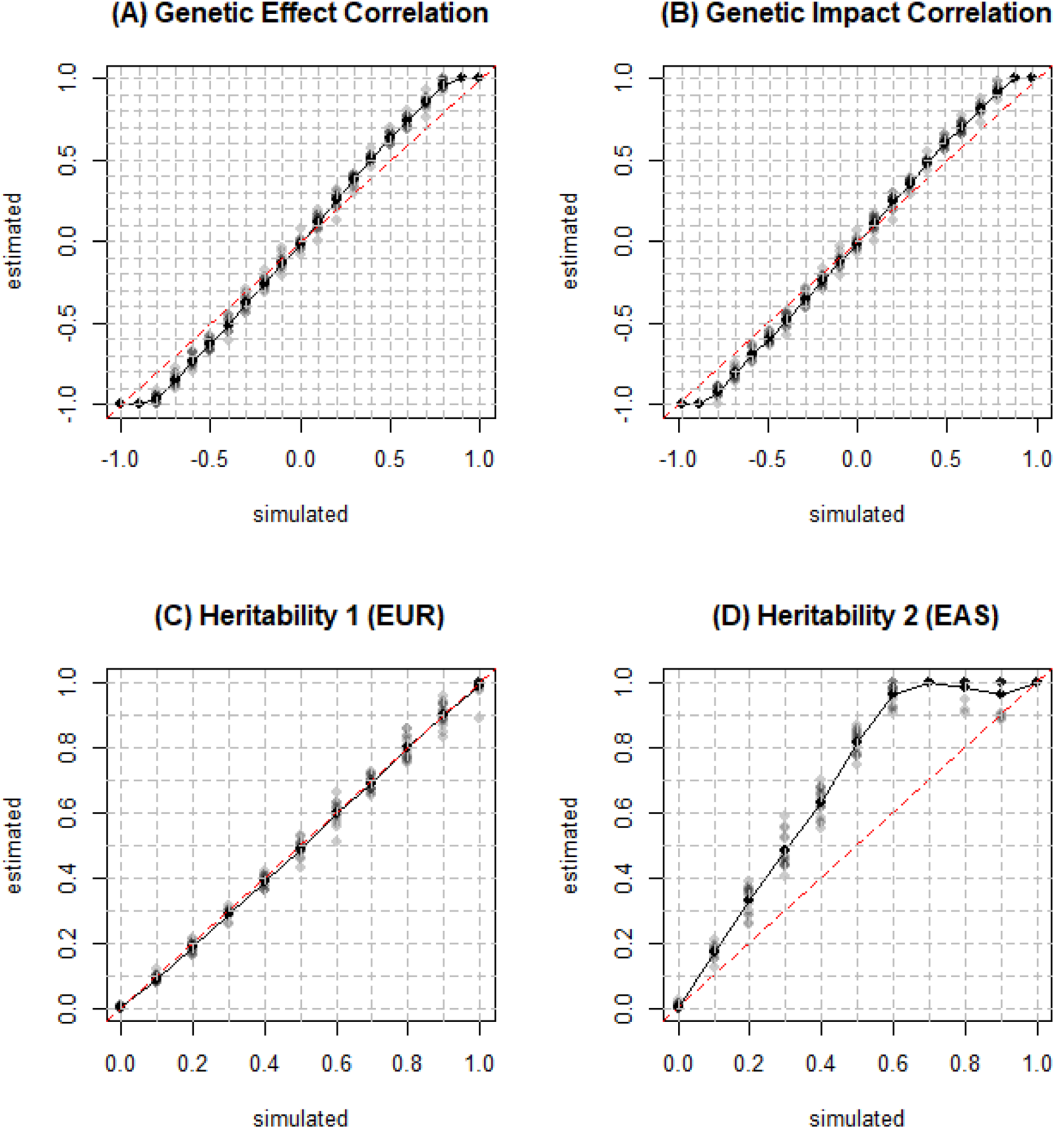
True and estimated genetic correlation and heritability for EUR and EAS with YRI to serve as external reference panel for EAS. Simulations were conducted on 26,119 simulated EUR and 20,147 simulated EAS individuals with 329,382 SNPs. The default genetic correlation and heritability was 0.5

For the summary statistics in pair 1, we chose 500 individuals randomly from YRI to serve as external reference panel for EAS. The correct reference panel was used for EUR. As shown in Fig. 5 the genetic correlation was overestimated by 20%. Heritability of EUR was accurate as expected and the EAS was overestimated by 60%. Inaccurate external reference panel has more dramatic influence on heritability compared with that on genetic correlation.

### Admixed population involved

**Fig. 6:**
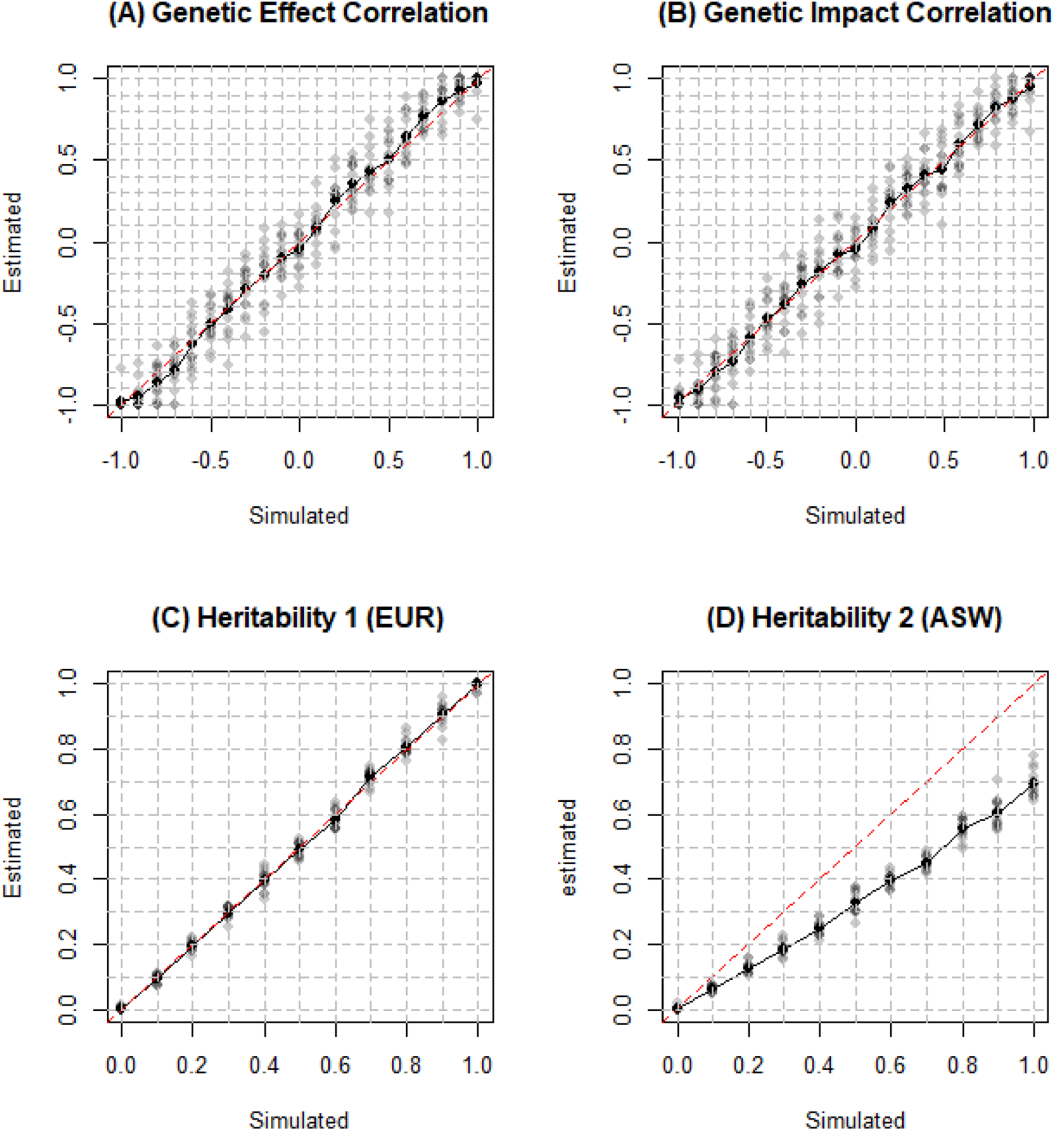
True and estimated genetic correlation and heritability for EUR and ASW. Simulations were conducted on 26,119 simulated EUR and 32,421 simulated ASW individuals with 238,164 SNPs. The default genetic correlation and heritability was 0.5

After filtering out SNPs with minor allele frequency below 1% and related individuals, we had 26,119 EUR individuals, 32,421 ASW individuals and 238,164 SNPs in the analysis. The results are shown in Fig. 6. Genetic correlation has been overestimated by 7%. Heritability of EUR was accurately estimated as expected since the external reference panel for EUR is appropriate and EUR is homogenous population while heritability of ASW has been underestimated by 25%. *Popcorn* cannot be applied to admixed population since it fails to account for long-range LD induced by admixed populations and take covariate effects into consideration.

**Fig. 7:**
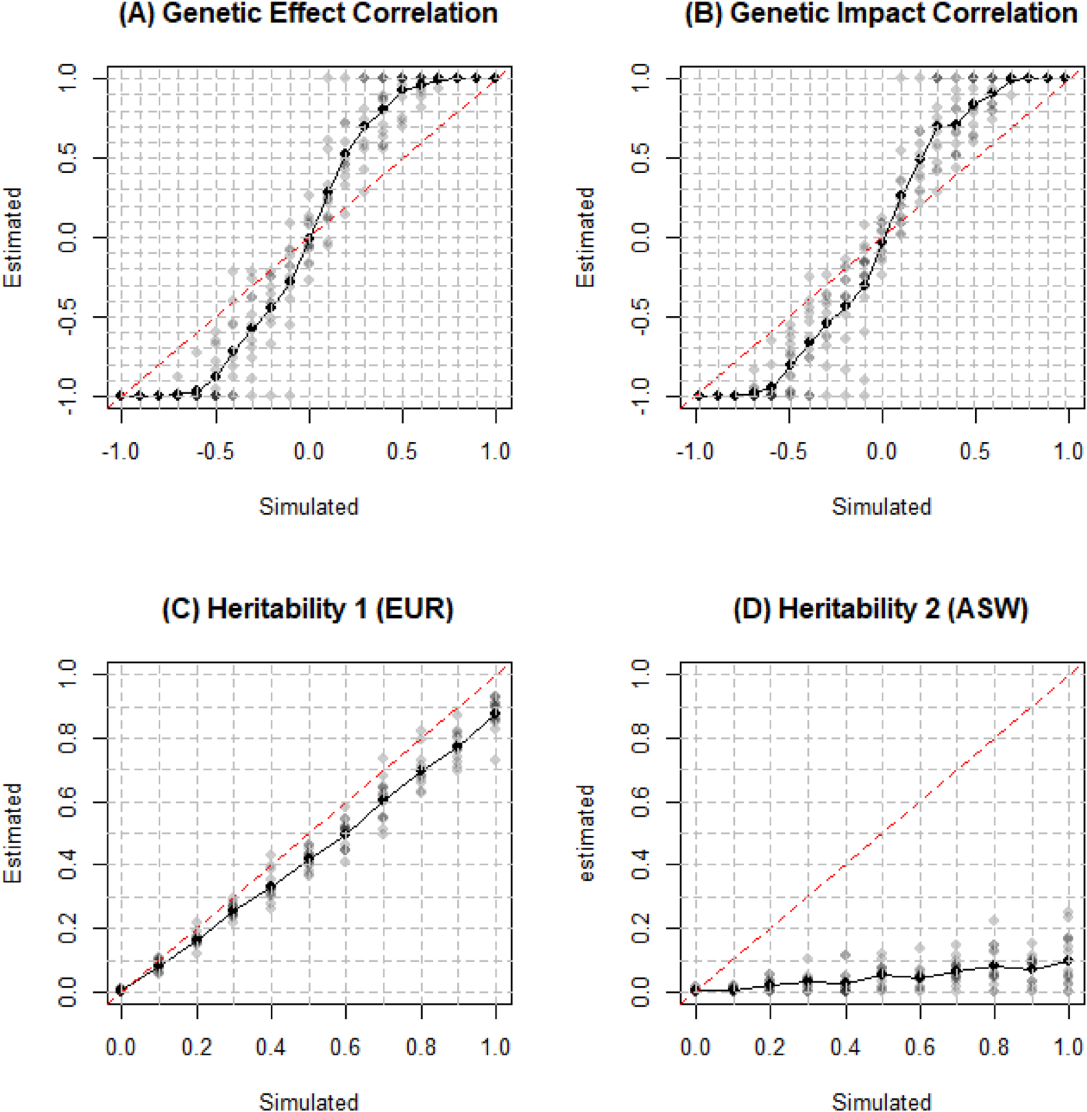
True and estimated genetic correlation and heritability for EUR and ASW with YRI to serve as external reference panel for ASW. Simulations were conducted on 26,119 simulated EUR and 32,421 simulated ASW individuals with 238,164 SNPs. The default genetic correlation and heritability was 0.5

We also tested how *Popcorn* performed with AFR serving as the external reference panel for ASW. As we could see in Fig. 7, the genetic correlation was even more overestimated, up to 80%. The situation of heritability of ASW was also worse. It was underestimated by 90%. Although African is one of the ancestries of African American, African population is not appropriate to serve as reference panel to estimate LD structure of African American in estimation of genetic correlation and heritability in *Popcorn* method.

## Discussion

In this work, we evaluated *Popcorn* under several input parameters and circumstances. We confirmed that 300–500 individuals in the external reference panel are able to provide adequate information for LD structure estimates. When the sample size reaches 5000–7000 and the number of SNPs 80,000–180,000, *Popcorn* is capable to generate accurate and stable estimates for both genetic correlation and heritability. We obtained similar conclusions from other ancestral population pairs though there was slightly different minimum number of sample size, number of SNPs and number of individuals in external reference panel. Moreover, we confirmed that misrepresentation of the reference panel overestimated the genetic correlation by 20% and heritability by 60%. Lastly, we found that the method overestimates the genetic correlation by 7–9% and underestimate the heritability by 25% with 39% more of SE when admixed population involved. The conclusions above are drawn according to the definition of ‘ideal estimates’ and should be modified if there is stricter or looser standardization. It provides plenty of valuable reference information for researchers using the method to obtain more accurate and stable estimates for both genetic correlation and heritability.

We used simulated data to do the evaluation both because we lacked real data results and simulation has multiple advantages: (1) we could change conditions and parameters as we want and expect the results; (2) we could use a small dataset to speed up the process to save time; and (3) we could produce large datasets, which is rare in real life, to do the test. However, the results and conclusion could be biased for real data since all tests are performed on simulated data with ideal conditions which are not possible to completely represent real life. We would like to extend our evaluation applied to more real data sets and related results in the future.

Bias is produced when we apply *Popcorn* on the simulation of EUR and ASW. *Popcorn* fails to account for long-range LD induced by admixed populations and take covariate effects into consideration. Determining the genetic correlation of complex traits among admixed populations from summary statistics is challenging. However, African Americans have a higher incidence and mortality than other ancestral populations for several traits including several cancer types. Many results of various cancers for homogenous populations (Europeans and Asians) from GWAS have been reported in previous publications but limited sample size and results for African Americans and other admixed populations. If the genetic correlation of prostate cancer between those non-admixed populations and African Americans can be estimated, the transferability of the results from former to African Americans can be instrumental for avoiding additional tests and research. We expect to extend *Popcorn* to estimate genetic correlation for admixed populations. A possible direction is to capture the complex LD and long-range correlations which was neglected before though adjusting principle components of SNPs as covariates into the calculation of LD scores (Luo et al. 2020).

## Supplementary Information

Below is the link to the electronic supplementary material.

Supplementary file1 (DOCX 17573 KB)

## Data Availability

Codes for simulation tests can be found at https://github.com/juzhang9/Popcorn_Eval. The datasets supporting the current study have not been deposited in a public repository because they were all generated from simulations descripted in Material and Methods part but are available from the corresponding author on request.
