# Supplementary material for: Evaluating the estimation of genetic correlation and heritability using summary statistics
Source: Mol Genet Genomics. 2021 Sep 29;296(6):1221–34. doi: 10.1007/s00438-021-01817-7 (PMC8550643; doi:10.1007/s00438-021-01817-7)
Supplement: Supplementary file 1 — Supplementary file1 (DOCX 17573 KB) [file 438_2021_1817_MOESM1_ESM.docx]

# Description of Supplemental Data

Supplemental Data include 12 figures.


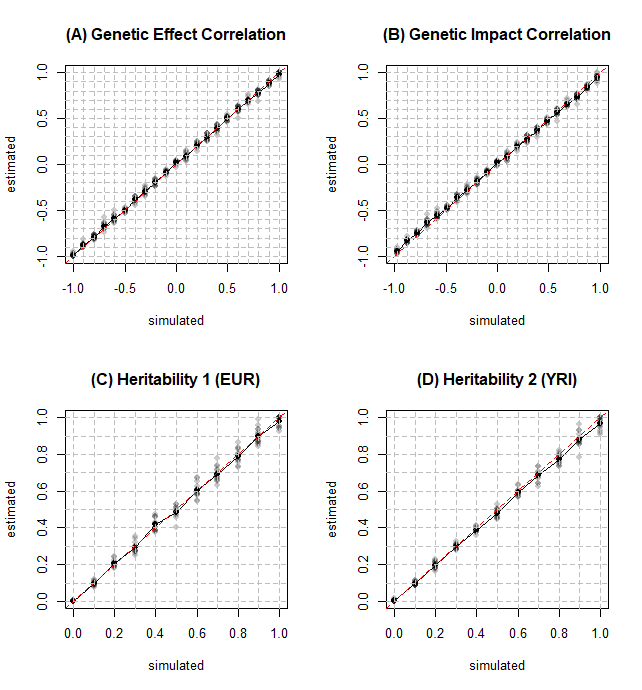


**Figure S1. True and estimated genetic correlation and heritability for EUR and YRI**

Simulations were conducted on 26,119 simulated EUR and 35,267 simulated YRI individuals with 220,687 SNPs. The default genetic correlation and heritability was 0.5.


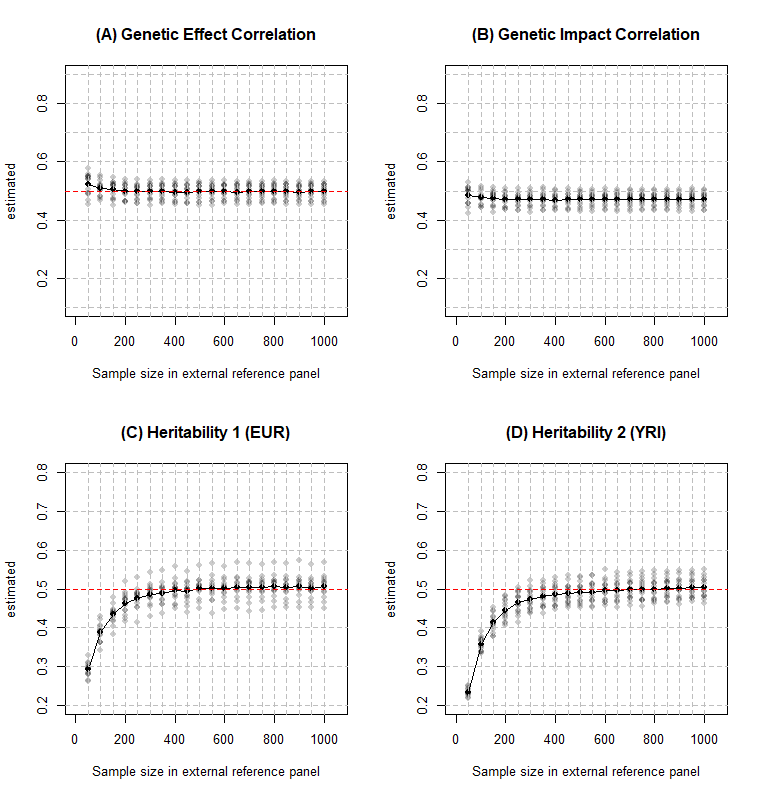


**Figure S2. Estimated genetic correlation and heritability for EUR and YRI from different sample size in external reference panel**

Simulations were conducted on 26,119 simulated EUR and 35,267 simulated YRI individuals with 220,687 SNPs. The default genetic correlation and heritability was 0.5.


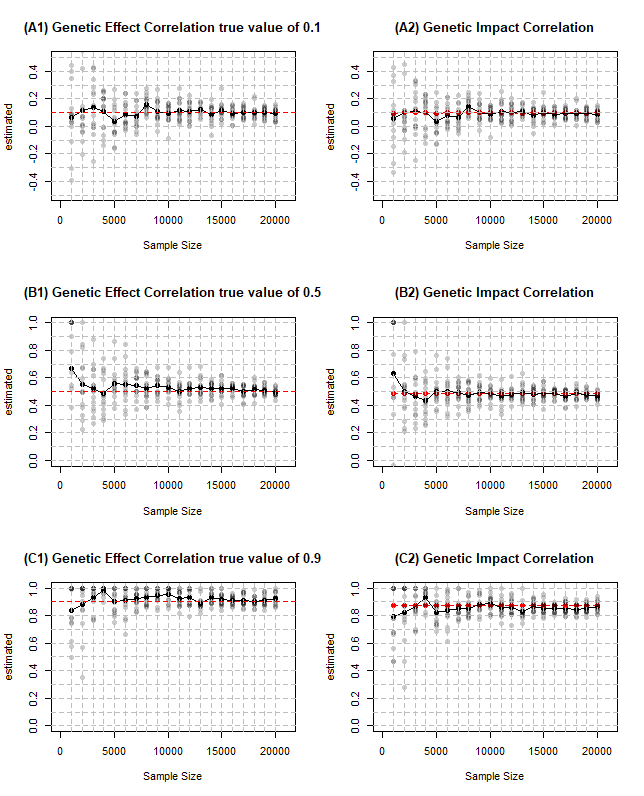


**Figure S3. Estimated genetic correlation between EUR and YRI from different sample size**

Simulations were conducted on different number of individuals with 220,687 SNPs and 500 individuals in external reference panel. The default heritability was 0.5 and genetic correlation was 0.1, 0.5 and 0.9 respectively in each row.


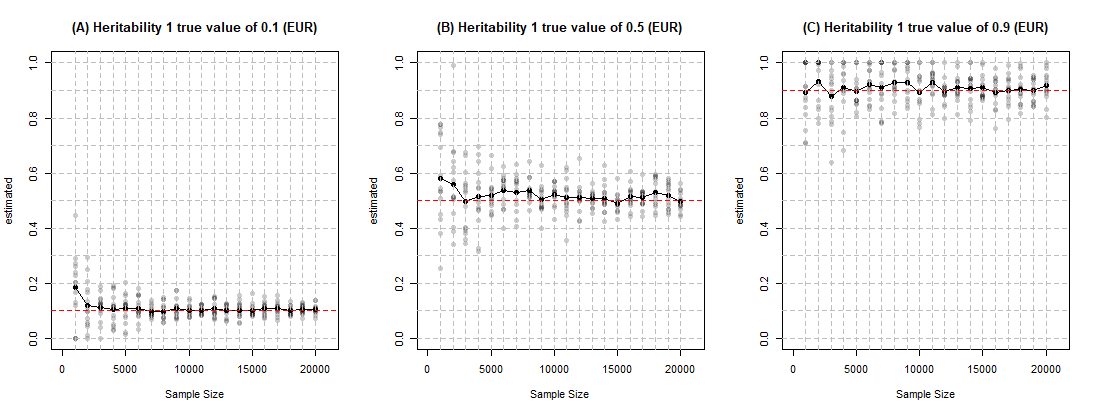


**Figure S4. Estimated heritability of EUR in population pair EUR and YRI from different sample size**

Simulations were conducted on different number of individuals with 220,687 SNPs and 500 individuals in external reference panel. The default genetic correlation was 0.5 and heritability was 0.1, 0.5 and 0.9 respectively.


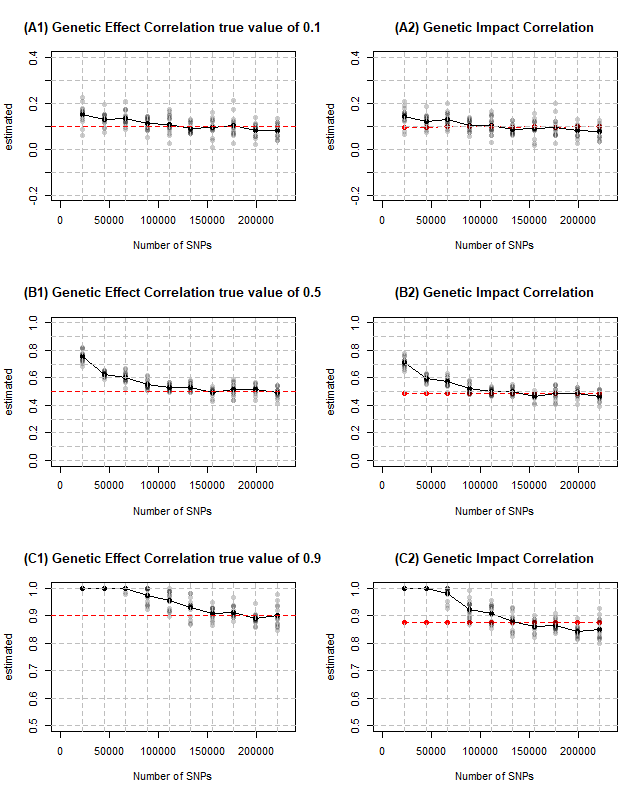


**Figure S5. Estimated genetic correlation between EUR and YRI from different number of SNPs**

Simulations were conducted on 26,119 simulated EUR and 35,267 simulated YRI individuals with different number of SNPs and 500 individuals in external reference panel. The default heritability was 0.5 and genetic correlation was 0.1, 0.5 and 0.9 respectively in each row.


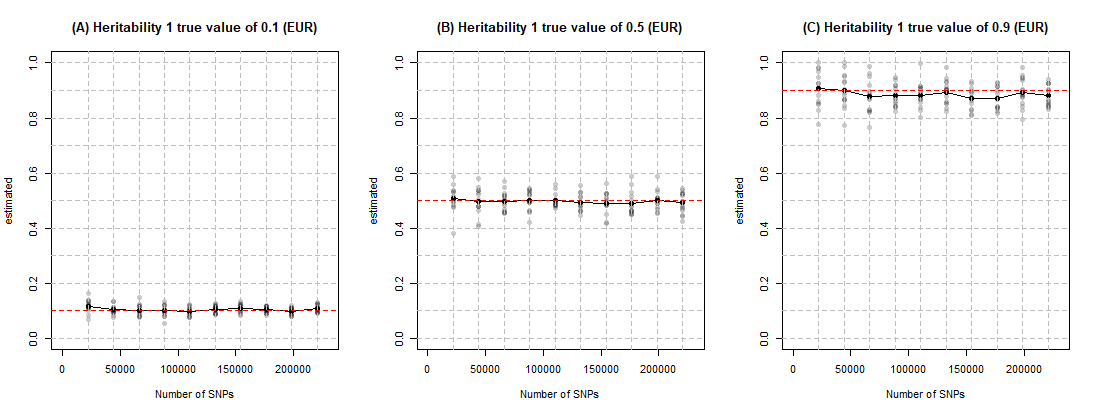


**Figure S6. Estimated heritability of EUR in population pair EUR and YRI from different number of SNPs**

Simulations were conducted on 26,119 simulated EUR and 35,267 simulated YRI individuals with different number of SNPs and 500 individuals in external reference panel. The default genetic correlation was 0.5 and heritability was 0.1, 0.5 and 0.9 respectively.


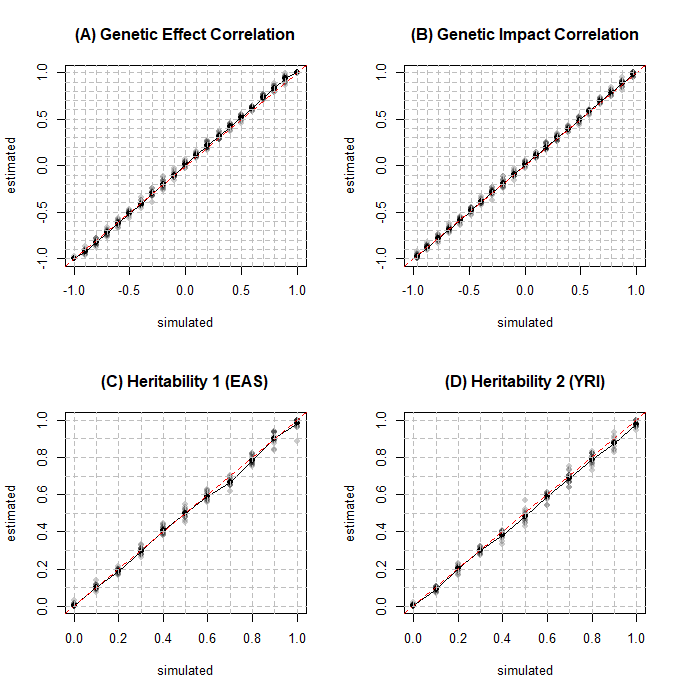


**Figure S7. True and estimated genetic correlation and heritability for EAS and YRI**

Simulations were conducted on 20,147 simulated EAS and 35,267 simulated YRI individuals with 186,726 SNPs. The default genetic correlation and heritability was 0.5.


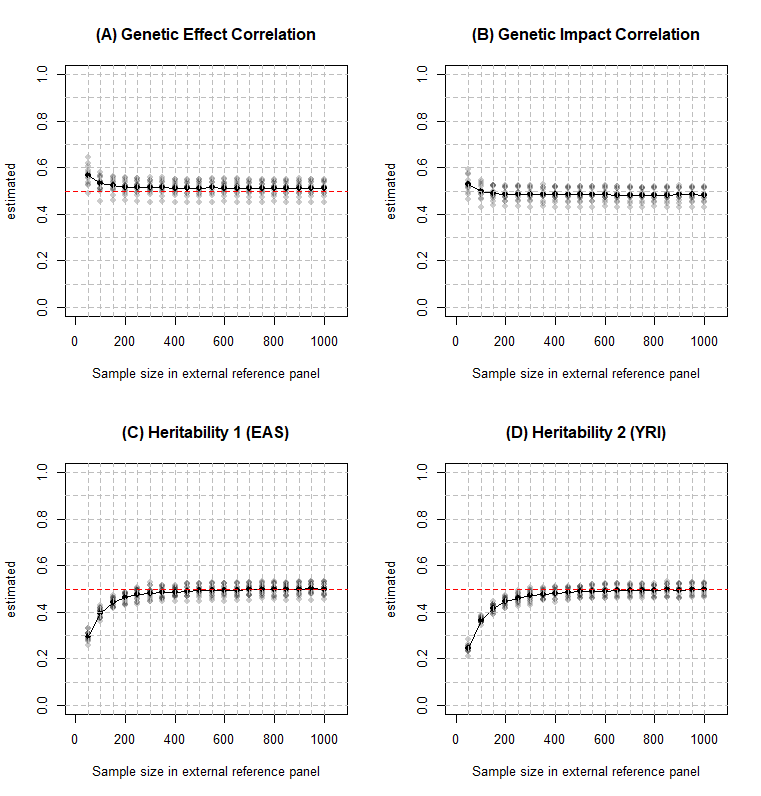


**Figure S8. Estimated genetic correlation and heritability for EAS and YRI from different sample size in external reference panel**

Simulations were conducted on 20,147 simulated EAS and 35,267 simulated YRI individuals with 186,726 SNPs. The default genetic correlation and heritability was 0.5.


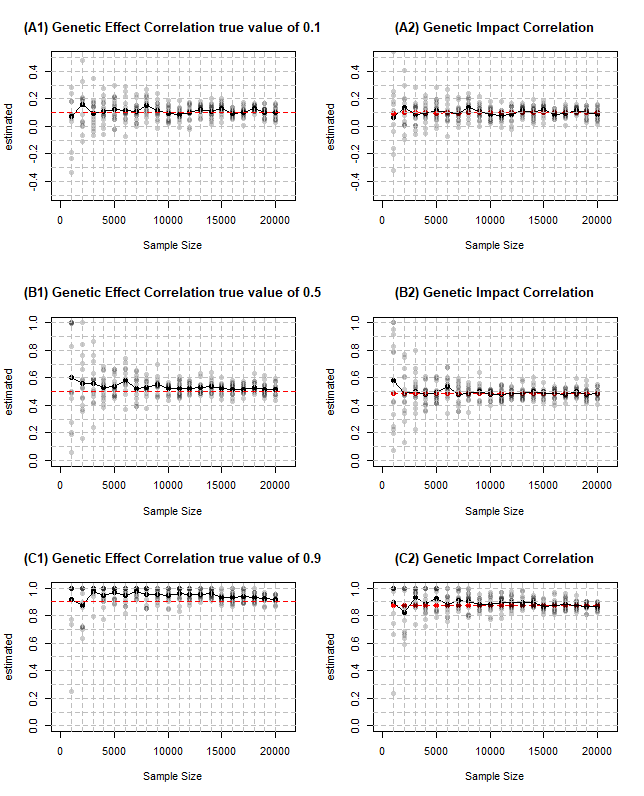


**Figure S9. Estimated genetic correlation between EAS and YRI from different sample size**

Simulations were conducted on different number of individuals with 186,726 SNPs and 500 individuals in external reference panel. The default heritability was 0.5 and genetic correlation was 0.1, 0.5 and 0.9 respectively in each row.


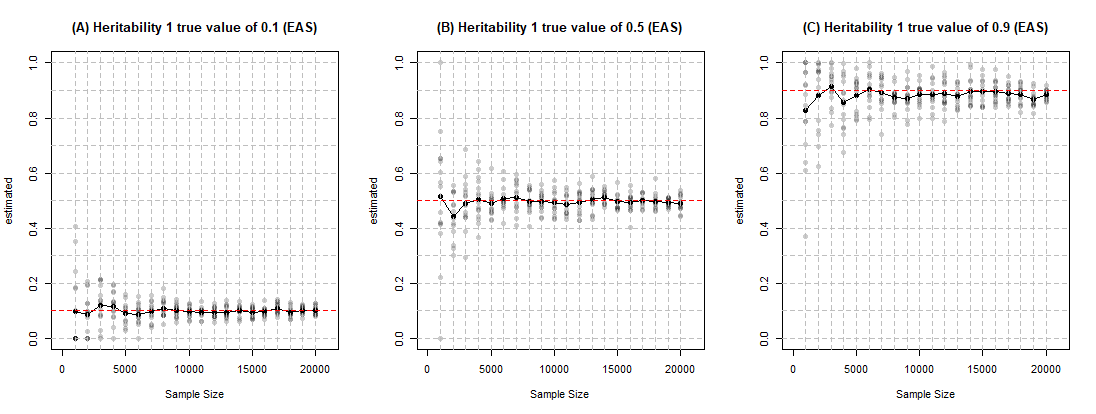


**Figure S10. Estimated heritability of EAS in population pair EAS and YRI from different sample size**

Simulations were conducted on different number of individuals with 186,726 SNPs and 500 individuals in external reference panel. The default genetic correlation was 0.5 and heritability was 0.1, 0.5 and 0.9 respectively.


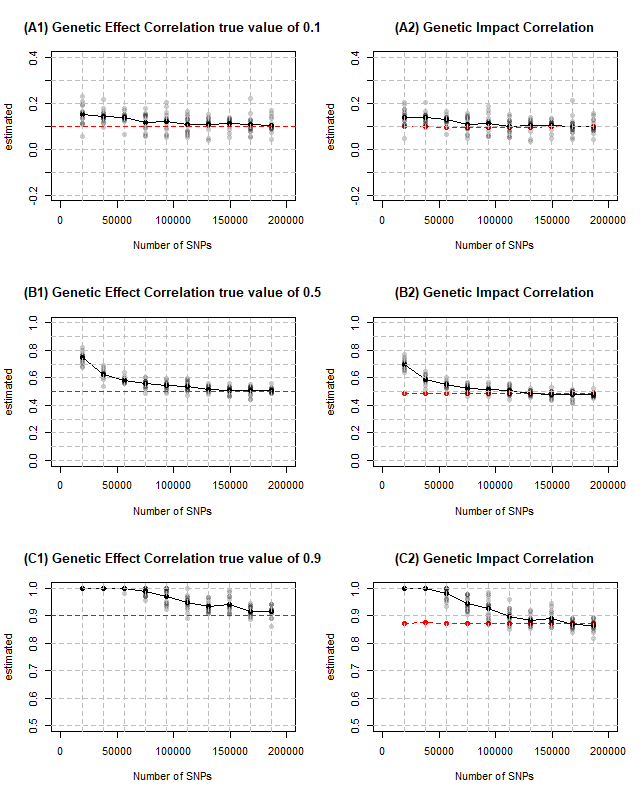


**Figure S11. Estimated genetic correlation between EAS and YRI from different number of SNPs**

Simulations were conducted on 20,147 simulated EAS and 35,267 simulated YRI individuals with different number of SNPs and 500 individuals in external reference panel. The default heritability was 0.5 and genetic correlation was 0.1, 0.5 and 0.9 respectively in each row.


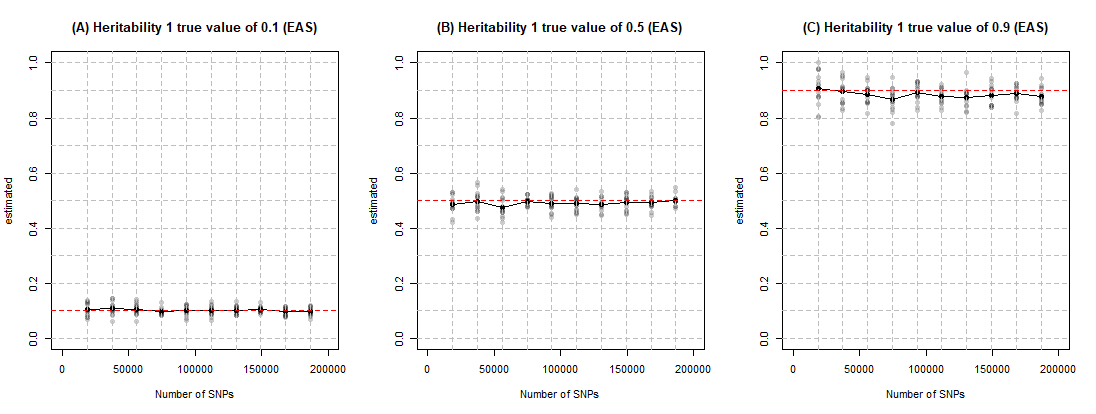


**Figure S12. Estimated heritability of EAS in population pair EAS and YRI from different number of SNPs**

Simulations were conducted on 20,147 simulated EUR and 35,267 simulated YRI individuals with different number of SNPs and 500 individuals in external reference panel. The default genetic correlation was 0.5 and heritability was 0.1, 0.5 and 0.9 respectively.
